# Cognitive impairment and depression in patients with relapsing–remitting multiple sclerosis depending on age and neuroimaging findings

**DOI:** 10.1186/s41983-021-00376-3

**Published:** 2021-09-08

**Authors:** Oksana O. Kopchak, Tetiana A. Odintsova

**Affiliations:** Department of Neurology, Psychiatry and Physical Rehabilitation, Kyiv Medical University of UAFM, Boryspilska 2, Kyiv, Ukraine

**Keywords:** Multiple sclerosis, Cognitive impairment, Depression, MRI

## Abstract

**Background:**

Multiple sclerosis is an insidious, disabling, both physically and mentally, demyelinating disease of the central nervous system. This work aims to evaluate relationships between cognitive impairment in separate domains, depression and their correspondence with MRI-findings, as well as the influence on each other’s manifestations, in patients with relapsing–remitting multiple sclerosis.

**Results:**

Visual–spatial/executive functions and memory domains suffered more frequently than others in the study subjects under 40 years; in patients over 40 years old memory, visual–spatial/executive functions and abstract thinking impairment prevailed the most. Such cognitive domains as memory, language, abstract thinking, visual–spatial and executive functions were impacted in both groups of patients even without the apparent cognitive decline according to MoCA scale. Presence of depression impacted language and attention more prominently than the rest of the domains only in participants younger 40 years. According to the MRI, frontal lobe, corpus callosum and periventricular area were affected more often compared to other brain regions in case of cognitive impairment; meanwhile, combined lesions of frontal lobe and corpus callosum, fronto-temporal region were associated with depression.

**Conclusion:**

Cognitive impairment and depression are one of the common, yet disabling and socially disrupting manifestations of MS. Quite frequently such complaints are neglected or considered as parts of comorbidities. At the same time cognitive impairment can be amplified by depression, especially in patients under 40 years.

## Background

Multiple sclerosis (MS) is a multifaceted chronic inflammatory autoimmune neurodegenerative disease of the central nervous system (CNS), which relatively rapidly leads to a severe long-term disability of a significant number of working-age population (2.8 million people worldwide) due to the impact of the neurological and motor deficit along with cognitive impairment (CI) and psycho-emotional disorders [1, 2]. Conventional magnetic resonance imaging (MRI) of the brain and the spinal cord plays the crucial role for diagnostic (according to the 2017 reviewed McDonald criteria) and disease-modifying purposes since it corresponds to the observed clinical picture [3].

Cognitive as well as neuropsychological deficit can be predicted already on preclinical stages (clinically isolated syndrome (CIS) and radiologically isolated syndrome (RIS)) by the localization of lesions. The most frequent MR findings connected with the presence of CI are lesions of corpus callosum (CC), fronto-temporal zone, subcortical areas, precuneus, thalamus and brain atrophy in general [4]. Brain atrophy in case of MS is far above the physiological loss of brain volume due to aging (0.5–1.35% per year) [5], and is already present on early stages of MS but can be partially reversed by disease-modifying therapy (DMT) [6]. Early MR-changes that can predict the future non-motor signs are decreasing of the hippocampus volume, as they are associated with gray matter atrophy over time [7].

Depression is a widespread comorbidity in patients with multiple sclerosis and is associated with increased disease burden and negative influence on the quality of life [8]. It was found that presence of depressive disorder in patients with MS is associated with damage of the fronto-limbic connections: the bilateral anterior thalamic radiation, cingulum, superior longitudinal fasciculus and uncinate fasciculus [9]. Therefore, depending on the localization of demyelination and axonal damage, CI and psycho-emotional disorders in those patients can accompany motor and neurological disorders, be present separately or in combinations [10]. However, the relationships between CI, depression and MR-findings, as the associations between one another, in relapsing–remitting MS (RRMS) patients are not studied to the full extent.

The aim of our study was to assess relationships between CI in separate domains, depression and their association with MRI-findings, as well as the influence on each other’s manifestations, in patients with RRMS.

## Methods

The current study enrolled 106 patients with RRMS (81 females and 25 males) aged from 22 to 67 years (mean age: 41.8 ± 10.7, disease duration (DD): 10.3 ± 8.5 years). According to the age, all the patients were divided into two groups: A—under 40 years (*n* = 48) and B— ≥ 40 (*n* = 58). Mean disease duration in group A was 6.25 ± 5.32 and in group B—13.5 ± 9.2. The study subjects were diagnosed RRMS according to McDonald’s criteria 2017 [3]. A medical history was obtained from every participant. The examination was composed of a standard clinical evaluation, neurological examination, the application of neuropsychological questionnaires, laboratory tests (complete blood count, biochemical parameters, TSH) and polymerase chain reaction test for Covid-19 (everyone had negative results). Each participant underwent MRI scan of brain. Siemens 1.5-T MRI Scanner with MAGNETOM Avanto SQ system was used for the study. In order to measure brain volume, the following parameters were evaluated: bifrontal index, caudal index, width of lateral ventricle in coronal plane, width of lateral ventricle in parasagittal plane, diameter of 3rd ventricle in coronal plane, diameter of 4th ventricle in coronal plane, ventricular index, subarachnoid spaces (cranio-cortical width, sino-cortical and interhemispheric width), gray matter (cortical) thickness in different areas. The disability level in MS patients was evaluated by means of Kurtzke’s Expanded Disability Status Scale (EDSS). Mild disability equals 1–3.5 points, moderate—4–6 points and 6.5–8 stand for severe disability [11]. The Montreal Cognitive Assessment (MoCA) was applied to evaluate presence and severity of CI. The MoCA includes six subcategories according to the domains: memory (M), language (L), attention (A), abstract thinking (AT), visual–spatial and executive functions (VS/EF). The scale score was interpreted as: 30–26 points—no CI; 25–18 points—mild CI; < 18 points—severe CI [12]. Beck Depression Inventory (BDI) was applied to find the presence and assess the severity of depression. The scale consists of 21 items that tap major depression symptoms in accordance to diagnostic criteria listed in the Diagnostic and Statistical Manual for Mental Disorders. Each answer is scored from 0 to 3 points. Mean score 0–9 indicates absence of depression, 10–18—mild depression, 19–29—moderate depression and 30–63—severe depression [13]. All participants were screened for the level of education.

The participants were excluded from the study if they were younger than 18, had progressive forms of MS, stage of exacerbation of RRMS or severe disability (EDSS score: 6.5—8 points), severe depression, sphincter disorders (urine incontinence, imperative urges to urinate and urine retention), pregnancy, cerebrovascular pathology or risk factors (stroke of any origin, arterial hypertension, obesity, dyslipidemia, diabetes mellitus), as well as patients treated with corticosteroids or INF-β at the time of the study, which could alter the study’s parameters.

All the study subjects provided written informed consent and the study was approved by the Institutional Ethics Committee.

All statistical data were processed by means of Graph Pad Prism version 9. Student’s *t*-test (*t*) was applied for evaluating credibility between mean quantitative positions of two samples. Proportions were compared using χ^2^. Relationships between different indicators were assessed using the Pearson’s correlation coefficient (*r*) according to statistical distribution. A *p* < 0.05 value was considered statistically significant.

## Results

Our participants had the following complaints: decrease in memory performance, difficulties in verbalization (vocabulary), inability to concentrate, decreased occupational performance, fatigue, lack of energy during usual daily activities, presence of disturbing thoughts, mood swings, lack of motivation, decreased mood. During the neurological examination pyramidal signs, pathological reflexes, increased muscle tone, impaired coordination (intention tremor, gait and truncal ataxia, missing the mark), brainstem disorders (vertigo, nausea, V and VII cranial nerves impairment), sensory disorders (in particular, impaired vibration and proprioception sense, paresthesia, Lhermitte’s sign) were revealed (Table 1).

**Table 1:** The incidence of neurological deficit among the patients of both groups (n, %)

The neurological deficit	Group A (*n* = 48)	Group B (*n* = 58)	*p*
Pyramidal disturbances	26 (54)	38 (65)	0.2343
Cerebellar dysfunction	18 (37)	22 (38)	0.9637
Brainstem disorders	13 (27)	20 (36)	0.4128
Sensory impairment	33 (69)	42 (72)	0.6798
Visual disorders	21 (44)	30 (52)	0.4134

Concerning the results of the brain MRI, the majority of the patients had multifocal lesions in the white and gray matter, especially in periventricular, CC, cortical (FL, TL, PL) areas, presence of BA (Table 2).

**Table 2:** Distribution of affected brain areas in both groups (*n*, %)

The affected area	Group A (*n* = 48)	Group B (*n* = 58)	*p*
Frontal lobe	31 (65)	42 (72)	0.3861
Temporal lobe	29 (60)	25 (43)	0.0759
Parietal lobe	11 (24)	30 (52)	0.0024
Occipital lobe	0 (0)	3 (5)	0.1099
Corpus callosum	30 (64)	43 (74)	0.1977
Brain atrophy	10 (21)	26 (45)	0.0094
Periventricular zone	44 (92)	55 (96)	0.5142
Cerebellum	27 (56)	31 (53)	0.7730
Brainstem	19 (39)	25 (43)	0.7143
Frontal lobe + brain atrophy	7 (15)	20 (36)	0.0193
Frontal lobe + corpus callosum	22 (46)	20 (36)	0.2343
Temporal lobe + corpus callosum	21 (44)	23 (40)	0.6702
Parietal lobe + corpus callosum	13 (27)	19 (34)	0.5264
Temporal lobe + parietal lobe	15 (31)	11 (19)	0.1434
Frontal lobe + temporal lobe + parietal lobe	10 (21)	8 (15)	0.3366
Temporal lobe + brain atrophy	7 (15)	10 (17)	0.7105
Parietal lobe + brain atrophy	9 (19)	15 (26)	0.3838

According to the MRI-findings, lesions of the PL, BA and combination of FL and BA were more frequently found in patients of the group B compared to the participants of the group A (*p* < 0.05) (Table 2).

Mean MoCA score in group A was 23.65 ± 4.53 and in group B—23.2 ± 3.7. Based on MoCA score, participants in both groups were divided into three subgroups: I—without CI (AI (*n* = 21); BI (*n* = 19)), II—with moderate CI (AII (*n* = 19), BII (*n* = 30)) and III—with severe (A III (*n* = 8), BIII (*n* = 9).

**Fig. 1:**
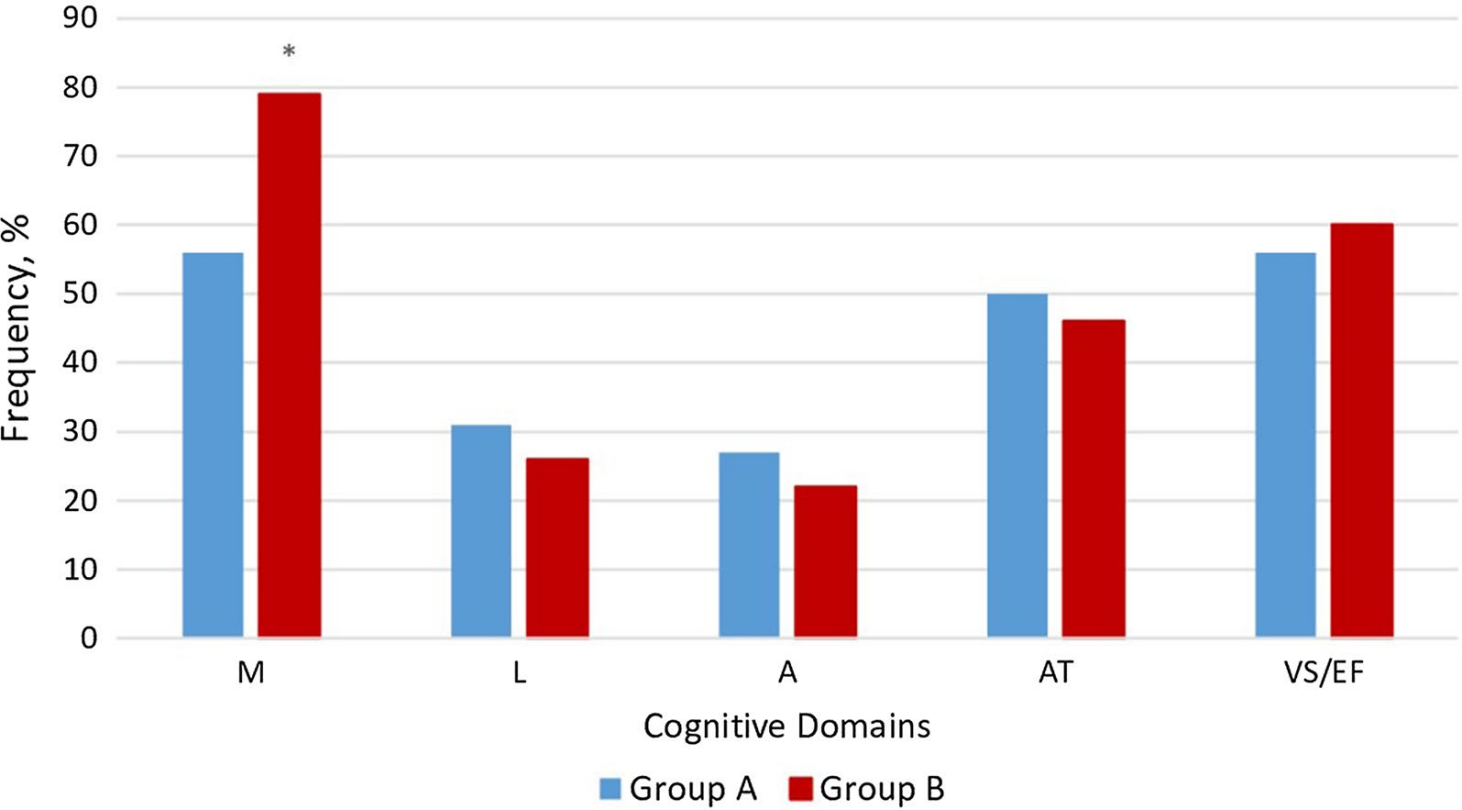
Distribution of affected domains in participants of both groups depending on age (*p = 0.0107)

In the A group VS/EF and M (both were present in 56% of participants) and AT (50%) domains were affected significantly more frequently than others; as for the group B, the most common deteriorated cognitive domains were: M (79%), VS/EF (60%) and AT (46%). The group B patients had more frequently deteriorated M domain in comparison with the group A (Fig. 1).

**Fig. 2:**
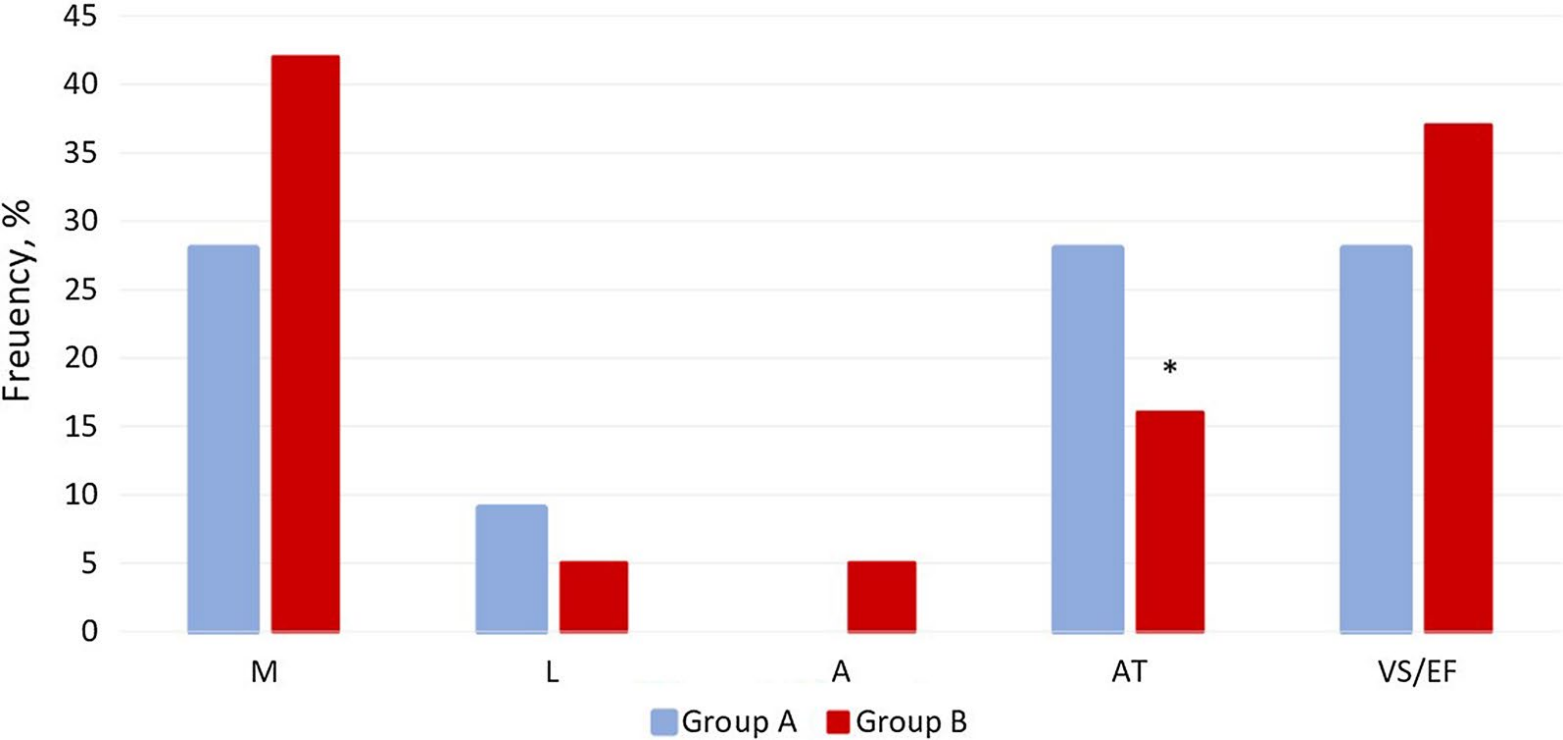
Distribution of affected domains in both groups depending on age in absence of CI (*p < 0.005)

It is remarkable that some domains were impacted without the apparent CI according to MoCA scale. There was a noticeable decline of attention in the group B, although no changes were found in this domain in the group A. Meanwhile, abstract thinking was more prominently deteriorated in the group A compared to the group B (Fig. 2).

In case of frontal lobe (FL) lesions VS/EF domain was more frequently impaired in AIII (100%) compared to AII (77%) (*p* = 0.0039) and AI (30%) (*p* = 0.0244); L domain was more widely affected in A III (100%) than in AII (23%) (*p* = 0.0010). Temporal lobe (TL) lesions were associated with M impairment that was more substantially expressed in group AIII (100%) in comparison with AII (69%) (*p* = 0.0131) and AI (23%) (*p* = 0.0183); VS/EF domain deterioration more frequently found in AII (77%) than in AI (31%) (*p* = 0.0183). Parietal lobe (PL) lesions were characterized by VS/EF impairment to a greater extent expressed in AIII (100%) than in AII (86%) (*p* = 0.0106) and AI (25%) (*p* = 0.0048) accordingly. Lesions of the corpus callosum (CC) negatively influenced on the memory more prominently in group AIII (100%) compared to AII (77%) (*p* = 0.0244) and AI (25%) (*p* = 0.0048); VS/EF—more frequently in AIII (100%) than in AII (62%) (*p* = 0.0048) and AI (30%) (*p* = 0.0025); AT—to a greater extent in AIII (100%) than in AII (54%) (*p* = 0.0466). Combined lesion of both FL and CC resulted in impairment of memory more prominently in AIII (100%) compared to AI (40%) (*p* = 0.0180); language was more affected in AIII (100%) than in AII (20%) (*p* = 0.0011); attention was impaired more often in AIII (86%) compared to AI (20%) (*p* = 0.0228); abstract thinking was more affected in AIII (86%) compared to AI (20%) (*p* = 0.0038); VS/EF more expressed in AIII (100%) than in AI (20%) (*p* = 0.0038). Lesions of both FL and TL were characterized by deterioration of AT more frequently in AII (70%) compared to AI (16%) (*p* = 0.0389). Injury of both FL and PL were accompanied by L dysfunction more pounced in AIII (100%) than in AII (20%) (*p* = 0.0098). Simultaneous lesions of FL, TL, PL and CC featured in the impairment of both M and VS/EF more expressed in AIII (100%) in comparison with AII (75%) (*p* = 0.0143).

**Fig. 3:**
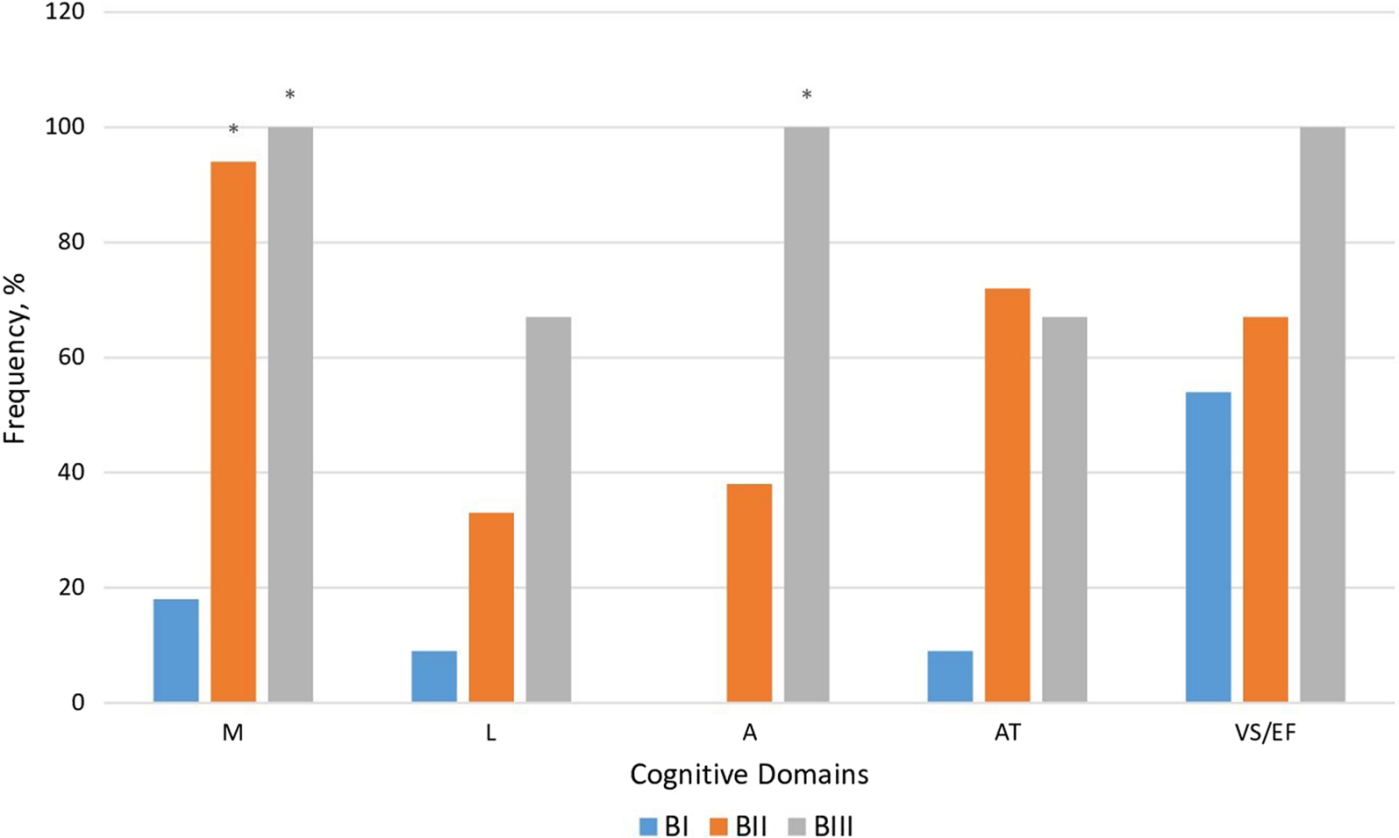
Distribution of affected domains in the B group depending on age, level of CI and simultaneous lesions of FL and CC (*p < 0.005)

As for the group B, lesions of the FL were characterized by changes in the following domains: memory, that was more significantly declined in BIII (100%) in comparison with BII (95%) (*p* = 0.0008) and BI (27%) (*p* < 0.0001); VS/EF impairment more frequently found in BIII (87%) than in BI (40%) (*p* = 0.0286); A decline more considerably in BIII (75%) than in BII (32%) (*p* = 0.0381); L impairment more prominently expressed in BIII (87%) in comparison with BII (21%) (*p* = 0.0013). In the case of TL lesions, significant deterioration was observed in memory domain to a greater extent in BIII (100%) (*p* = 0.0350) and BII (100%) (*p* = 0.0004) compared to BI (33%). Lesions of PL negatively influenced on the following domains: memory impairment was more frequently expressed in group BIII (100%) (*p* = 0.0073) and BII (93%) (*p* = 0.0032) in comparison with BI (25%); VS/EF changes—more prominently manifested in BII (92%) than in BI (42%) (*P* = 0.0094); L impairment—more frequently in BIII (83%) compared to BII (33%) (*p* = 0.0455). Lesions of CC were associated with deterioration in such domains: M—more considerably in group BIII (100%) (*p* = 0.0050) and BII (95%) (p < 0.0001) than in BI (36%); L to a greater extent in BIII (87%) than in BII (32%) (*p* = 0.0125); attention more in BIII (57%) than in BII (18%) (*p* = 0.0198). Brain atrophy was accompanied by memory impairment more frequently in BIII (100%) (*p* = 0.0376) and BII (100%) *p* = 0.0031) compared to BI (44%); A deterioration, that was more prominently expressed in BIII (80%) (*p* = 0.0128) in comparison with BII (17%), nonetheless VS/EF dysfunction was to a greater extent found in BI (100%) than in BII (55%). Combination of brain atrophy and of FL lesions was characterized by: M decline more pronounced in BIII (100%) (*p* = 0.0133) and BII (100%) (*p* = 0.0022) compared to BI (28%); AT impairment to a greater extent in BIII (100%) as compared to BII (44%) (*p* = 0.0376); attention disorders that were more frequently expressed in BIII (80%) than BII (22%) (*p* = 0.0363). TL lesions combined with brain atrophy were marked by: memory deterioration more prominently in BII (100%) than in BI (33%) (*p* = 0.0350); L impairment—to a greater extent in BII (100%) comparing with BIII (80%) (*P* = 0.0164). Simultaneous lesions of FL and CC featured with memory deterioration that was more prominent in BIII (100%) (*p* = 0.0012) and BII (93%) (*p* < 0.0001), compared to BI (18%); attention was more prominently affected in BIII (100%) than in BII (38%) (*p* = 0.0121) (Fig. 3). In case of lesion of both FL and TL memory was the most frequently affected domain in BIII (100%) (*p* = 0.0164) and BII (100%) (*p* = 0.0024) comparing with BI (20%). Combination of lesions in FL and PL distinguished by memory deterioration, that was more considerably affected in BIII (100%) (*p* = 0.0037) and in BII (87%) (*p* = 0.0037) than in BI (27%); VS/EF dysfunction, which was more prominent in BII (89%) comparing with BI (45%) (*p* = 0.0428); learning impairment to a greater extent expressed in BIII (83%) than in BII (22%) (*p* = 0.0428). Simultaneous lesions of the FL, TL and PL were characterized by the following changes: memory deterioration more frequently in BIII (100%) (*p* = 0.0020) and BII (100%) (*p* = 0.0050) than in BI (9%); VS/EF impairment, that was to a greater extent in BII (100%) in comparison with BI (27%) (*p* = 0.0241).

**Fig. 4:**
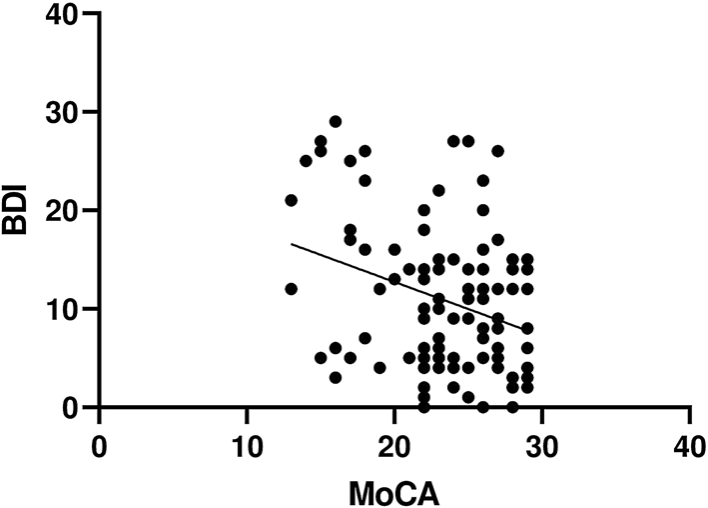
The regression relationship between CI and depression severity in the group A

Considering the results of BDI, the study subjects had either no signs of depression or its presence of mild and moderate severity, as the severe level was not detected. In group A 23 (48%) patients had no signs of depression, 19 (40%) had mild depression, 6 (12%) had moderate. As for the group B: 29 (50%) had no depression, 19 (33%)—mild depression, 10 (17%)—moderate one. Significant negative correlation between CI and depression severity was found in the group A of patients (*r* = − 0.3717, *p* = 0.0093), indicating that CI can influence the depressive disorder and vice versa (Fig. 4). No significant correlation was found between CI and depression severity within the subgroups of the A group.

Combination of lesions of FL and CC demonstrated the following results: 12 (54%) patients had no depression, 5 (23%)—mild, 5 (23%)—moderate depression. Such combination in addition demonstrates presence of negative correlation between CI and depression severity (*r* = − 0.4807, *p* = 0.0235). In case of lesions in FL and TL 11 (58%) patients did not have depression, 5 (27%)—mild and 3 (15%) had moderate, there was a relevant negative correlation between depression and CI severity (*r* = − 0.4704, *p* = 0.0421). Combined lesions of FL and PL were accompanied by absence of depression in 5 (36%) patients, mild depression—in 6 (43%) participants, moderate—in 3 (21%) patients, that had a significant negative correlation with CI severity (*r* = − 0.5488, *p* = 0.0421). In the case of TL and PL involvement, 6 (40%) participants did not have depression, 7 (47%)—mild, 2 (13%)—moderate, there was a significant negative correlation between CI and depression severity (*r* = − 0.5848, *p* = 0.0220). Lesions of TL and CC showed the following results: 10 (48%) patients were without depression, 8 (38%)—with mild and 3 (14%)—with moderate; a significant negative correlation between CI and depression severity was found (*r* = − 0.4688, *p* = 0.0321). Combination of PL and CC lesions resulted in such findings: 5 participants (38%) had no depression, 5 (38%)—mild and 3 (24%)—moderate, there was negative correlation with CI severity (*r* = − 0.5590, *p* = 0.0470). Brain atrophy was associated with absence of depression in 3 (30%) patients, mild depression—in 4 (40%), moderate—in 3 (30%) patients; negative correlation was found between CI and depression severity (*r* = − 0.8068, *p* = 0.0048). In this group depression influenced on the two cognitive domains in a greater amount than the rest: L (*p* = 0.0469) and A (*p* = 0.0060).

As for the group B: 29 patients (50%) had no depression, 19 (33%)—mild depression, 10 (17%)—moderate one, though this group did not reveal any correlation with CI severity in general as well as in separate subgroups depending on the MRI-findings. Combination of FL and CC lesion was characterized by absence of depression in 14 (31%) participants, mild in—13 (29%) and moderate—in 18 (40%). Considering lesions of FL accompanied by brain atrophy, 10 patients (48%) did not have depression, 6 (28%)—mild and 5 (24%)—moderate. Combination of lesions in FL and TL were accompanied by the following results: 7 participants (41%) had no signs of depression, 6 (35%) had mild and 4 (24%)—moderate. Combined lesions of FL and PL discovered that 13 (50%) had no depression, 9 (35%) had mild and 4 (15%)—moderate. In case of lesions in TL and PL 7 participants (64%) had no depression, 3 (27%)—mild and 1 (9%)—moderate. Lesions of TL and CC showed the following results: 11 (55%) did not have depression, 7 (35%) had mild, 2 (10%)—moderate. Lesions of PL and CC revealed that 13 patients (57%) had no signs, 7 (30%)—mild and 3 (13%)—moderate depression. In case of brain atrophy: 13 participants (54%) had no signs of depression, 6 (23%) had mild and 5 (23%)—moderate. The presence of depression showed no connection with disease duration in the both groups, implying that its development for the most part depends on the brain damage.

Presence of mild and moderate depression in group A was detected predominantly in the case of combined lesions of PL and CC as well as of FL and CC. As for the group B, presence of depression was mostly associated with such combinations of lesions: FL and CC; FL and TL. In accordance with the results of our study, presence of depression proved to impact CI severity in the group A, even considering separate brain regions, as well as in combination. Presence of brain atrophy was associated with strong negative correlation between CI and severity of depression only in the group A. No such relationship was observed in the group B, indicating that deterioration of CI and depression did not depend on one another’s level of severity in our study subjects over 40 years.

Among all participants 59 had higher education (29 did not have CI, 26 had moderate and 4 had severe) and 47 did not (11 had no signs of CI, 27 had moderate and 9 had severe). Absence CI was more frequently diagnosed in those patients of the group A with high education (*p* = 0.0066). No correlation was found between CI and disease duration in both groups of patients.

## Discussion

Several previous studies established that appearance of CI on preclinical and early RR-MS stages can predict the future disease progression [14, 15]. In this study, we discovered that several cognitive domains were affected in patients in both groups without the CI according to MoCA scale, though we cannot state that these changes will lead to the future deterioration of MS in these individuals, its needs further observations. Our research revealed that frontal lobe, corpus callosum and periventricular area were affected more often compared to other brain regions, that is consistent with results of Migliore et al. [16], who declared that the frontal lobe was highly sensitive to its numerous connections with other cortical and subcortical structures, so it could be triggered by the damage of any of them and resulted in CI; as well as with the study of Du et al. [17], applying fractional amplitude of low-frequency fluctuation (fALFF) analysis achieved results that lesions of hippocampal area, dorsolateral prefrontal lobe, and orbitofrontal cortex are connected with CI in RR-MS patients, specifically indicating that hippocampal and dorsolateral prefrontal areas are associated with working memory, while the orbitofrontal cortex is responsible for motivation, sensorimotor processing, self-evaluation and mediating the interactions between emotional processes and cognitive functions. According to Trenova et al. [18], the most frequently impaired domains were attention, information processing speed, memory, executive functions, and visuospatial skills, therefore, we observed visuospatial, executive functions and memory declined more frequently in the patients under 40 years, whereas, memory, visuospatial, executive functions and abstract thinking took the lead in the study subjects above 40 years.

Brochet and Ruet [19] in their review, reported that disease duration highly impacted severity of the CI, indicating that it specifically influenced memory, language and abstract thinking, nonetheless, no substantial correlation between the level of CI and disease duration in the patients of both age groups was detected in our study.

Concerning the neuropsychological disorders, depression in particular, is associated with lesions and atrophy in fronto-temporal and frontal lobes separately [20]. Our study similarly confirmed that appearance of depression was connected to the lesions of parietal lobe, corpus callosum, simultaneous of frontal lobe and corpus callosum (in group A) and combined lesions of frontal lobe and corpus callosum, fronto-temporal region (in group B), although brain atrophy was associated with depression only in the group of study subjects younger than 40.

It was established that depression was the most widespread psychiatric disorder known to potentially impact cognitive performance in MS patients, especially information processing speed, executive function, attention, motor function, and memory, but they might affect virtually all cognitive domains [14, 21–23], although Whitehouse et al. [21] stated that MS participants of their study with depression had reduced cognitive performance, but the working memory was intact. However, our findings demonstrated a negative correlation between CI and depression severity only in patients younger than 40 years and for the most part it influenced language and attention; no such correlation was revealed in participants over 40 years.

Considering presence of higher education, it was stated to have favorable effect on MS patients’ cognitive functions [24]; therefore, our study revealed higher prevalence of cases with intact cognition and substantially lower number of severe cognitive impairment in participants with higher education.

## Conclusions

Cognitive impairment and psycho-emotional disorders, specifically depression, are the one of the common, yet disabling and socially disrupting manifestations of MS. Quite frequently such complaints are not properly screened or are considered as parts of comorbidities. At the same time, cognitive impairment can be impacted by depression especially in case of patients younger than 40 years. Results of our study emphasize that all MS patients require a mandatory thorough screening of cognitive impairment and depression on early stages in order to prevent further socio-economical maladjustment and disability of such patients and enroll them in adequate disease-modifying therapies.

## Data Availability

The datasets used and/or analyzed during the current study are available from the corresponding author on reasonable request.

## Abbreviations

MS: Multiple sclerosis
RR-MS: Relapsing–remitting multiple sclerosis
CNS: Central nervous system
CI: Cognitive impairment
MRI: Magnetic resonance imaging
RIS: Radiologically isolated syndrome
CIS: Clinically isolated syndrome
FL: Frontal lobe
TL: Temporal lobe
PL: Parietal lobe
CC: Corpus callosum
DMT: Disease-modifying therapy
DD: Disease duration
EDSS: Expanded Disability Status Scale
MoCA: Montreal Cognitive Assessment
M: Memory
L: Language
A: Attention
AT: Abstract thinking
VS/EF: Visual–spatial and executive functions
BDI: Beck depression inventory
